# Serum soluble receptor of advanced glycation end products and risk of metabolic syndrome in Egyptian obese women

**DOI:** 10.17179/excli2017-275

**Published:** 2017-06-29

**Authors:** Moushira Zaki, Sanaa Kamal, Shams Kholousi, Hala T. El-Bassyouni, Walaa Yousef, Hanaa Reyad, Ramy Mohamed, Walaa A. Basha

**Affiliations:** 1 Biological Anthropology Department, National Research Centre, Cairo, Egypt; 2 Immunogenetics Department, Human Genetics and Genome Research Division, National Research Centre, Giza, Egypt; 3 Clinical Genetics Department, National Research Centre, Cairo, Egypt

**Keywords:** metabolic parameters, advanced glycation end products, RAGE, obese women

## Abstract

Obesity is one of the diagnostic criteria of metabolic syndrome (MS). It is correlated with insulin resistance (IR) and high vascular risk as well. Advanced glycation end products (AGEs) and their receptor (RAGE) play an important role in abnormal metabolic components in obese women. This study aimed to explore the serum levels of sRAGE in Egyptian obese women and compare with healthy non-obese controls and investigate the relationship between serum sRAGE, metabolic parameters, and obesity complications. The soluble form of receptor for advanced glycation end products (sRAGE), anthropometry, metabolic and biochemical biomarkers were measured in 100 obese women and 100 age-matched healthy control non-obese women. The homeostasis model assessment estimate of insulin resistance (HOMA-IR) has been determined from serum insulin and glucose values. Serum sRAGE levels were significantly lower in obese cases than controls and inversely correlated with obesity and metabolic parameters. Results of univariate and multivariate analyses for determinants of serum sRAGE levels in obese cases showed that parameters statistically and significantly related were body mass index (BMI), waist circumference (WC), LDL-C, TG, BP, HOMA-IR, ALT and AST. sRAGE is a novel biomarker for metabolic dysfunction in Egyptian obese women and might predict the future cardio-metabolic events.

## Introduction

Metabolic syndrome along with its all clinical manifestations is highly associated with the development of chronic diseases. Advanced glycation end products (AGE) are formed by non-enzymatic glycoxidation of proteins and peptides after exposure to aldose sugars (Schmidt et al., 1994[25]; Singh et al., 2001[28]). AGEs accumulate in the vessel walls and cause alteration of the extracellular matrix, cell surface receptors, or could even affect the function of intracellular proteins (Singh et al., 2001[28]). Interactions between AGE and its receptors (RAGE) provoke oxidative stress and stimulate pro-inflammatory or pro-coagulant cellular responses that comprising increases in the vascular cell adhesion molecule-1 and tumor necrosis factor-alpha expression (Brownlee, 1995[5]).

RAGE is a multiligand receptor of the immunoglobulin superfamily that employs diverse ligands relevant to the pathogenesis of atherosclerosis (Koyama et al., 2005[17], 2007[18]). RAGE has a C-truncated secreted isoform, designated soluble RAGE (sRAGE). In contrast to the cell surface RAGE, sRAGE is acting as a decoy where it blocks cell surface RAGE ligand binding and its consequent signaling (Hudson et al., 2005[15]). Reduced levels of sRAGE have been described as an important novel biomarker in patients with hypertension, type 2 diabetes, and also in non-diabetic subjects with coronary artery disease (Geroldi et al., 2005;[9] Falcone et al., 2005[7]). Some studies have also determined that levels of plasma sRAGE are negatively associated with the components of MS, body mass index (BMI), waist-to-hip ratio, serum triglycerides (TG), systemic arterial pressure, and insulin resistance index in the adult population (Koyama et al., 2005[17]; Norata et al., 2009[23]). Accordingly, sRAGE, obesity, and MS are strongly associated with atherosclerosis. The progression of atherosclerosis commences during adolescence or even earlier, however, few studies have examined the relationship between sRAGE, obesity, and MS among adolescents (He et al., 2014[11]). In adults, an inconsistency has been observed between studies that examined the relation between sRAGE and metabolic syndrome components (Yamagishi et al., 2006[32]; Norata et al., 2009[23]; Hudson et al., 2014[14]). Hence, our study enrolled 100 obese women and 100 non-obese women to investigate the relationship between serum sRAGE, metabolic parameters and obesity complications.

## Methods

### Subjects

One hundred obese women between ages of 18 and 35 years were recruited from the obesity clinic, National Research Centre. None of the study participants had diabetes according to the criteria of American Diabetes Association (Puavilai et al., 1999[24]). We excluded women who smoked cigarettes, taking medication that affect lipids or hormones, with cardiovascular disease or chronic kidney or liver disease, pregnant or breastfeeding, or had any other major illness. One hundred age-matched healthy non-obese women are included in the present study as a control group. Written informed consent was gained from each woman after a complete description of the study. The research has been authorized by the Ethical Committee of National Research Centre, Egypt (number = 16361), in accordance with the World Medical Association's Declaration of Helsinki. 

### Statistical analysis

We performed the statistical analyses using SPSS16.0 for Windows (SPSS Inc). The distribution of the variables was examined using Kolmogorov-Smirnov test of normality to verify whether it is followed a Gaussian pattern. Means ± SDs were used in order to express the normally distributed data, while logarithmic transformation was performed to all skewed variables. These variables, including triglycerides, fasting plasma glucose, and serum sRAGE levels, were presented as median and interquartile ranges. The unpaired t-test or the Mann-Whitney U test was used, as appropriate, to evaluate differences between the two groups of continuous variables. Categorical variables are presented by frequency counts, and intergroup comparisons were analyzed by a chi-square test. Simple (univariate) and multiple (multivariate) logistic regression analyses were performed to determine the associations between studied parameters and MS. Two-tailed P<0.05 was considered statistically significant.

### Clinical and biochemical assessment

All patients and controls were subjected to full medical history and clinical examination. BMI has been determined as weight in kilograms divided by height in meters square (kg/m^2^). Waist circumference (WC) and hip circumference (HC) have been measured in cm using a plastic, non-stretchable tailor's tape. Subsequently, the Waist-to-Hip-Ratio (WHR) was calculated as WC divided by HC. Skin-fold thickness was measured in all subjects at the biceps, triceps, subscapular, supra-iliac, and abdominal areas using Holtain caliper then, the sum of skin folds was calculated. A full description of the anthropometric measurements has been reported elsewhere (Zaki et al., 2015[33]). All measurements were obtained according to standardized equipment and following the recommendations of the International Biological Program (Hiernaux and Tanner, 1969[12]). Body fat % was assessed by Tanita Body Composition Analyzer (SC-330).

Blood pressure was measured according to a standardized operating procedure using calibrated sphygmomanometer and brachial inflation cuff (HEM-7200 M3, Omron Healthcare, Kyoto, Japan). Systolic and diastolic blood pressures (SBP and DBP) were measured twice in the right arm in a sitting position after a 10-min rest period, then the average is used for analysis. Hypertension was defined as SBP ≥ 130 mmHg and/or DBP ≥ 85 mmHg or the use of antihypertensive medications.

### Laboratory measurements

Venous blood samples were collected by direct venipuncture after an overnight fast (minimum 12 h). Fasting plasma glucose and serum lipids (total cholesterol, high-density lipoprotein cholesterol (HDL-C), triglycerides (TG) were measured by enzymatic colorimetric methods using a Hitachi auto-analyzer 704 (Roche Diagnostics, Switzerland) (Hirschler et al., 2010[13]). Low density lipoprotein cholesterol (LDL-C) was calculated according to certain equation (LDL-C= Total cholesterol -Triglycerides/5+HDL-C). Serum insulin concentration was analyzed by chemiluminescent immunoassay (Immulite 2000, Siemens, Germany) (Chu et al., 2006[6]). Insulin resistance has been estimated by the Homeostasis Model Insulin Resistance (HOMA-IR); as the outcome of fasting plasma insulin level (IU/mL) and fasting plasma glucose level (mmol/L) divided by 22.5 (Matthews et al., 1985[21]). Serum sRAGE was measured using a commercially available Quantikine ELISA kit (R&D System, Inc., Minneapolis, MN).

## Results

The basic characteristics of the study participants are shown in Table 1(Tab. 1). The concentration of serum sRAGE was significantly lower in obese cases than in controls. In addition, the obese patients had significantly higher levels of BMI, WC, WHR, BP levels, ALT, AST, total cholesterol, TG, LDL and HOMA-IR (p<.05).

We further analyzed the clinical and anthropometric parameters of obese women by quartiles of serum sRAGE levels (Table 2(Tab. 2)). The mean concentration of sRAGE was 1.355±505 pg/ml while it was <557 pg/ml for the lower quartile. Subjects in the lower quartile serum sRAGE showed significant higher values of BMI, WC, SBP, DBP, HOMA-IR, total cholesterol, TG and LDL-C, ALT and AST compared to the highest quartile. 

Table 3(Tab. 3) shows univariate and multiple logistic regression analysis. Univariate analysis revealed that BMI, waist circumference, SBP, DBP, HOMA-IR, LDL-C,TG, ALT and AST were inversely associated with serum levels of sRAGE in obese women. After performing multivariate analyses, these parameters still remained significant and were independently related to serum levels of sRAGE including BMI > 30 (kg/m^2^),WC > 90 cm, hypertension, HOMA-IR > 3.3, LDL-C, TG, ALT and AST (R2 = 0.294).

## Discussion

In this study, we demonstrated that serum levels of sRAGE were associated with metabolic risk markers such as hypertension, central obesity and elevated lipid parameters among Egyptian obese women. Furthermore, an inverse relationship between sRAGE and HOMA-IR was also observed. The findings of our study are partially in agreement with a previous study (Norata et al., 2009[23]), in which sRAGE was found to be inversely associated with BMI and waist-to-hip ratio, no association with metabolic syndrome has been noticed though. According to the results of Norata and colleagues (2009[23]), the level of plasma sRAGE might reflect the disturbed metabolic status that could later cause vascular complications and diabetes preceding to any of the clinical complication. The results of the present study also are in agreement with the work of Basta et al. (2006[2]) which showed that serum sRAGE concentrations were downregulated in insulin resistance status. Our study suggests that circulating endogenous sRAGE levels may become a novel biomarker of abnormal metabolic status and lipid metabolism in obese women. Previously, serum sRAGE levels have been proposed as biomarkers of severity or prognosis of several diseases, and modified by several factors, such as BMI, RAGE polymorphisms, kidney dysfunction and some medications such as statin (Maillard-Lefebvre et al., 2009[20]).

The lower levels of sRAGE in obese subjects that have been noticed in our study were in agreement with the work of Brix and colleagues (2012[3]), who observed lower levels in obese cases compared to controls and an increase in sRAGE levels has been observed after bariatric surgery. Moreover, Vazzana and colleagues (2012[31]) observed that sRAGE significantly increases following diet-induced weight loss in obese women. However, another study conducted by Lorenzi and colleagues (2014[19]) showed contrast results, where higher levels of sRAGE have been reported in obese subjects and it went significantly lower after the gastric bypass surgery. They even suggested that restoring of several physiological functions that associated with gastric bypass and decreasing the metabolic syndrome could also have contributed to the rapid elimination of sRAGE of the body. It was suggested that these controversial findings are due to the inclusion/exclusion criteria applied (Lorenzi et al., 2014[19]). Bariatric surgery has also been shown to promote a reduction in oxidized low-density lipoprotein (LDL) (Garrido-Sánchez et al., 2008[8]). Lorenzi and colleagues (2014[19]) have reported improvements in the lipid profile, decreased LDL, increased HDL and diabetes attenuation following weight-loss surgery. It is well known that low HDL levels contribute substantially to higher vascular risk (Gordon et al., 1977[10]). 

The mechanism of interaction between sRAGE, obesity, and MS is not yet clear. As many ligands that interact with RAGE are important inflammatory regulators (Ueno et al., 2010[30]), it is likely that inflammation might be the linkage between sRAGE, obesity, and MS. A possible cellular mechanism for activating this inflammatory signaling has been suggested by Shoelson et al. (2006[27]). In addition, another study further illustrated that RAGE could modulate atherosclerosis via adiposity and could be involved in the progression of atherosclerosis in non-diabetic status (Ueno et al., 2010[30]). The recently higher prevalence of obesity is most probably a cause of the rising incidence of insulin resistance and MS, as well as CVD and type 2 diabetes. The possible role of sRAGE in the pathogenesis of obesity and MS has been shown in earlier life as well (He et al., 2014[11]). However, a contrasting data regarding the relationship of sRAGE with CVD risk and disease has been reported. Some studies showed a positive relationship between sRAGE and CVD in long-term diabetes (type 1 and 2) (Selvin et al., 2013[26]). On the contrary, an inverse association between sRAGE and CVD risk has been detected in general community-based cohorts (Hudson et al., 2011[16]). Further clinical and biological studies are required in order to explore the cause of these contrasting results and whether the role of sRAGE as a biomarker is affected by the presence of chronic disease such as diabetes. No influence of diabetes on the sRAGE levels has been reported though (Lorenzi et al., 2014[19]). There is a possibility that other factors might affect sRAGE levels and cause these controversial results. In addition to metabolic components, race, ethnicity and kidney function, there are other factors such as age, smoking and some types of medication including anti-diabetic medication, statins, ACE inhibitors have been shown to affect sRAGE levels (Tam et al., 2010[29])

The present study showed that lower circulating sRAGE concentrations may be associated with higher prevalence of metabolic risk parameters and increasing the prevalence of central obesity, elevated BP, LDL and TG among Egyptian obese women. In addition, an inverse association between sRAGE level and liver enzymes (ALT and AST) has been observed as well. It was proposed that metabolic syndrome might have non-traditional components such as subclinical inflammation, microalbuminuria, and non-alcoholic fatty liver disease (NAFLD) (Mulhall et al., 2002[22]; Adams et al., 2005[1]). Some mechanisms have been suggested to illustrate the associations of ALT and AST-to-ALT ratio with the metabolic syndrome. One of these proposed mechanisms is that these markers have been found to be positively correlated with hepatic fat content and NAFLD has harmful effects on metabolic syndrome components (Browning et al., 2004[4]). Hudson et al. (2014[14]) conducted a multi-ethnic community-based study and showed that lower sRAGE levels are associated with metabolic syndrome and its components that related to obesity, diabetes and hypertension. Although this association varies by ethnicity, the study emphasized the role of RAGE as a risk factor for metabolic and vascular disease (Hudson et al., 2014[14]).

In conclusion, our data showed an association between reduced sRAGE levels in obese subjects with high levels of lipid and metabolic markers. This association could raise the possibility that, the measurement of sRAGE level may improve risk assessment among obese women. This study supports the potential role of sRAGE in the development of poor clinical outcomes in obese women and might predict future cardio-metabolic events. Future studies should examine whether these differences in sRAGE levels have an implication for asymptomatic obese subjects.

## Acknowledgements

This work was supported by grant from National Research Centre, Egypt.

## Conflict of interest

The authors declare no conflict of interest.

## Figures and Tables

**Table 1:**
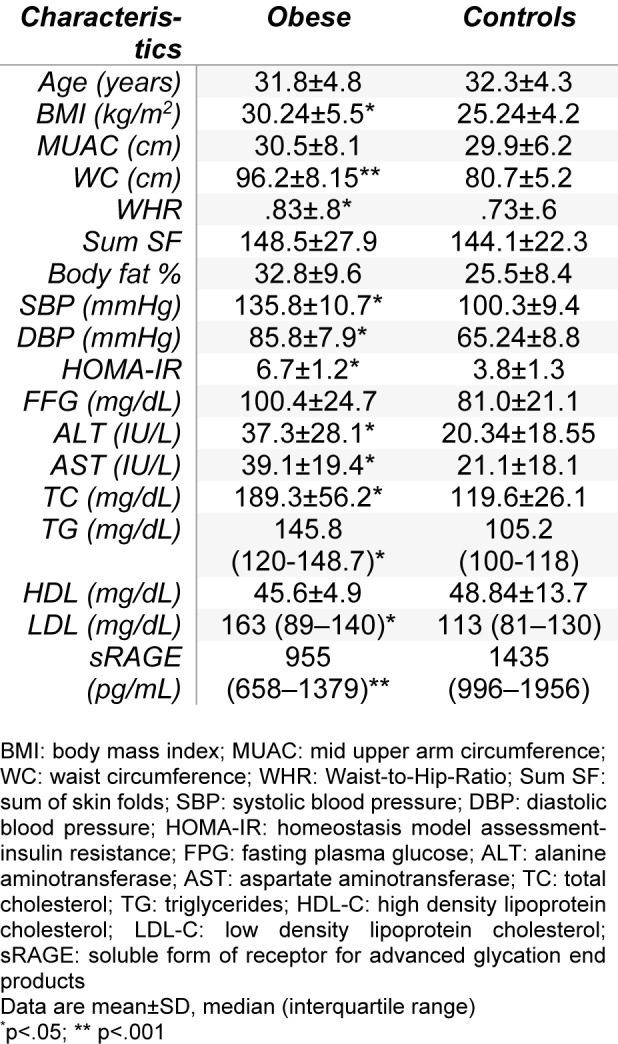
Clinical characteristics of obese women and control subjects

**Table 2:**
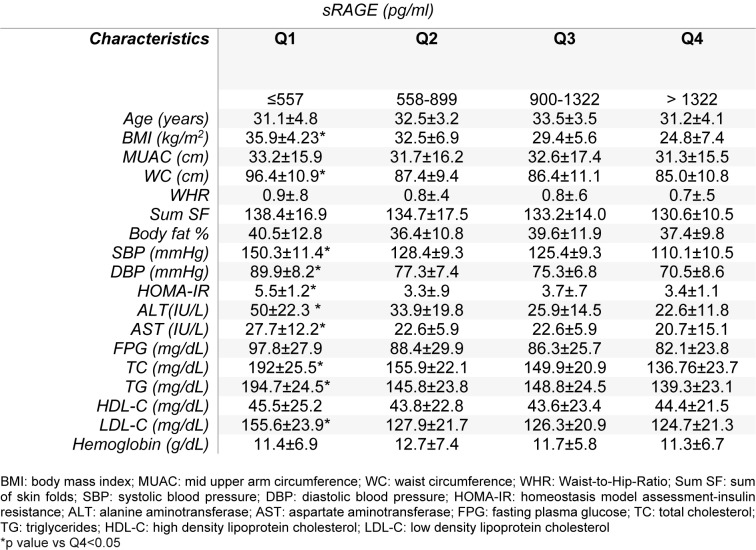
Clinical and anthropometric parameters of obese women, stratified according to quartiles of circulating sRAGE concentrations

**Table 3:**
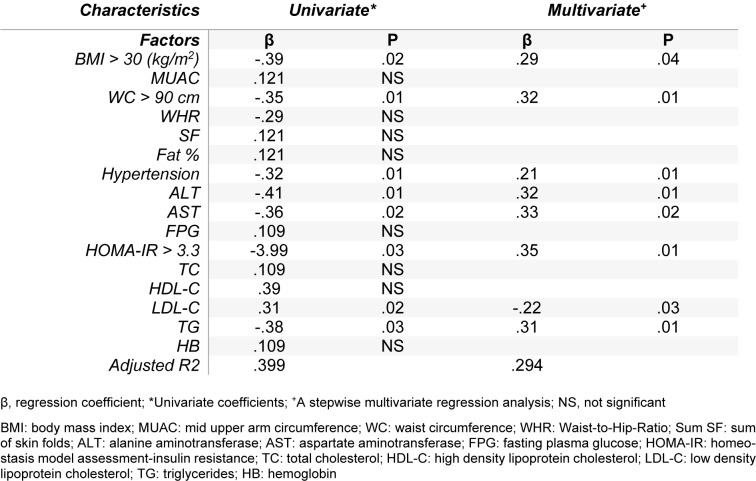
Univariate and stepwise multiple regression analyses for determinants of sRAGE in obese cases
